# The Beneficial Use of *Artemisia annua*, Artemisinin, and Other Compounds in Animal Health

**DOI:** 10.3390/ani15101359

**Published:** 2025-05-08

**Authors:** Estefania Morua, Laura Cuyas, Luis Matías-Hernández

**Affiliations:** R&D Department, Biotech Tricopharming Research SL, Carrer Pallars 108, 08018 Barcelona, Spain; em@tricopharming.com (E.M.); lcc@tricopharming.com (L.C.)

**Keywords:** animal health, *Artemisia annua*, artemisinin, cancer, coccidiosis, gut health, leishmaniasis, pharmacodynamic synergy, standardization

## Abstract

Plants and plant-derived natural products have been used in traditional medicine for centuries. The lack of effective therapies in the modern world to address several diseases, the increasing development of drug resistance, and the growing interest in herbal medicine have led to the study and resurgence of natural ancient remedies. *A. annua,* commonly known as sweet wormwood or sweet annie, is a medicinal plant widely known for its antimalarial properties. In the past decade, increasing evidence has demonstrated the plant’s broad therapeutic potential, including antitumoral, antimicrobial, antiparasitic, metabolic, and immunomodulatory effects, among others. While most research has focused on human health, there is growing interest in exploring the veterinary applications of *A. annua* and its bioactive compounds, particularly artemisinin. This review aims to summarize the current knowledge on the beneficial effects of *A. annua*, artemisinin, and other compounds in animal health. It also highlights the need for standardizing *A. annua* metabolites to ensure the reliability and efficacy of treatments and explores how artemisinin works in synergy with other molecules present in *A. annua*.

## Simple Summary

This article reviews the beneficial use of *Artemisia annua* and its primary active compound, artemisinin, in animal health. While the plant is widely known for its antimalarial properties, promising emerging research reveals a broader therapeutic potential. Both *A. annua* and the artemisinin biomolecule exhibit, among other properties, antiparasitic, immunomodulatory, anti-inflammatory, antioxidant, and potentially anticancer activities, which could have a significant impact on animal health. This constitutes the central point we explored throughout this review. Furthermore, this manuscript emphasizes the importance of standardizing the plant’s active metabolites to ensure both the efficacy and consistency of its therapeutic potential, while also exploring the synergistic interactions between artemisinin and other bioactive compounds within the plant. These findings position *A. annua* as a promising natural adjuvant in veterinary phytotherapy, particularly for managing conditions like coccidiosis, other gastrointestinal disorders, leishmaniasis, and certain cancers in animals.

## 1. Introduction

*A. annua* (also known as qinghao in Chinese) is a medicinal plant from the *Asteraceae* family, recognized for over 2000 years in traditional Chinese medicine for its therapeutic properties [1,2]. Its use is documented in the ancient text ‘Shennong Bencao Jing’, which compiles oral traditions describing medicinal plants and their applications. It is noted that *A. annua* was historically used to treat fever and is frequently cited in ancient herbals [1,2].

Over centuries, *A. annua* was used in traditional medicine, though its effectiveness was not fully understood until the 1960s–70s. During China’s Project 523, launched in 1967 to combat malaria, researchers explored both synthetic drugs and traditional remedies. A major breakthrough came from the latter, leading to the discovery of *A. annua*’s antimalarial properties. In 1972, a research team led by Tu YouYou successfully isolated artemisinin, a sesquiterpene lactone highly effective against *Plasmodium falciparum*, the parasite responsible for malaria. To confirm its efficacy, *A. annua* extracts were tested in mouse models and later in human clinical trials, showing promising results. Although the process was lengthy and costly, it culminated in one of the most significant medical breakthroughs of the 20th century, saving millions of lives and significantly impacting public health. In 2015, Tu YouYou was awarded the Nobel Prize in Medicine, highlighting not only the value of traditional medicine but also the importance of exploring natural resources for contemporary health solutions [3,4].

Today, *A. annua* and its active compound, artemisinin, produced exclusively in the glandular trichomes mainly on the plant’s leaves, remain the focus of extensive medical research. Scientists are exploring its potential for treating a wide range of diseases in both humans and animals. Beyond artemisinin, there is growing interest in the synergistic effects between artemisinin and other bioactive compounds present in the leaves of *A. annua*, such as flavonoids, phenolic acids, and polysaccharides. These secondary metabolites may enhance artemisinin’s pharmacodynamic efficacy by increasing its bioavailability, prolonging its activity, or modulating different cellular pathways [1,5].

Moreover, these other secondary metabolites also exhibit independent pharmacological properties, including anti-inflammatory, antioxidant, and antimicrobial activities, making *A. annua* a promising polypharmacological therapeutic candidate. Rather than focusing only on artemisinin, researchers are now exploring the broader medicinal potential of the entire plant. This integrated approach could open new avenues for combination therapies targeting malaria, cancer, viral infections, and autoimmune diseases [1,5,6,7].

The history of *A. annua* is a testament to the successful blend of ancient knowledge and modern science, demonstrating how a traditional remedy can evolve into a critical resource within the ”One Health” concept. This concept recognizes the interconnectedness of human, animal, and environmental health, highlighting the importance of integrated approaches in addressing health challenges and benefiting all living organisms [8,9].

In this context, *A. annua* has gained attention for its promising medicinal properties in addressing animal health challenges. Although research in this area is still developing compared to human health studies, this review serves as a starting point, focusing on conditions supported by substantial evidence in the existing literature.

## 2. Materials and Methods

To identify relevant scientific publications, a comprehensive literature search was performed using multiple online academic databases and platforms. These included PubMed, ResearchGate, ScienceDirect, Google Scholar, and SpringerLink. The search focused on publications related to both the biological activity and therapeutic potential of *Artemisia annua* and its compounds, particularly artemisinin, within the context of animal health.

Specific keywords used to perform the search included the following: *animal health*, *antioxidants*, *artemisinin*, *Artemisia annua*, *coccidiosis*, *gut microbiome*, *leishmaniasis*, *cancer*, and *pharmacodynamic synergy*. From this strategy, a total of 208 articles were identified and reviewed. These encompassed in vivo and clinical studies, as well as systematic reviews and mechanistic investigations, providing a strong and well-rounded base of information to support the development of this review.

## 3. *Artemisia annua* and Its Role in Supporting Gut Health

Gut health can be defined as a steady state where the microbiome and the intestinal tract exist in symbiotic equilibrium and where the welfare and performance of the animal are not constrained by intestinal dysfunction [10,11]. The gut microbiota is a highly diverse and dynamic community composed of over 10^14^ microorganisms, including bacteria, fungi, and viruses, which collectively work to preserve a stable intestinal environment [12]. This microbial ecosystem is unique to each host and evolves over time, influenced by internal factors such as genetics, epigenetics, age, immune system function, gut physiology, and hormonal regulation, as well as external factors, including diet, medications, and environmental exposures [10,13,14].

The gut microbiome plays a crucial role in nutrient absorption, metabolism, digestion, immune system modulation, and intestinal barrier maintenance. Beyond microorganisms, it comprises structural microbial components, metabolites, environmental factors, and host–microbe interactions, all of which contribute to maintaining gut health and overall well-being [14]. A balanced microbiome is essential for sustaining gastrointestinal health and its biodiversity is fundamental for metabolic processes, immune function, and disease resistance [14]. Disruptions in microbial diversity and functionality, known as dysbiosis, can lead to disorders such as diarrhea, inflammatory bowel disease (IBD), colitis, or systemic infections [15,16,17,18]. Additionally, dysbiosis has been associated with a range of health conditions, including cardiovascular, renal, and neurological diseases, as well as behavioral disorders [14,19,20,21,22].

Several strategies are employed to treat and prevent gut disorders in animals. Probiotics (live beneficial microorganisms that support gut health) and prebiotics (compounds that feed beneficial bacteria improving their growth or activity) play a key role in increasing resistance to pathogenic bacteria and enhancing mucosal immunity, thereby improving overall health [23]. Exogenous enzymes, particularly in poultry, are commonly used to enhance feed efficiency and nutrient absorption [24,25]. For specific conditions such as colitis, diarrhea, and coccidiosis, treatments may include antibiotics, anti-inflammatory medications, rehydration, and antiparasitic drugs. Additionally, there is growing interest in the use of medicinal plants to enhance productivity and to treat or prevent intestinal disorders [26,27,28].

One plant of particular interest is *A. annua* and its active compound, artemisinin, which have shown promising effects on modulating the gut microbiome and enhancing intestinal health across various species. *A. annua* supports gut health by enhancing beneficial bacteria and reducing pathogens. In geese, its inclusion increased *Bacteroides*, *Fecalibacterium*, and *Paraprevotella*, promoting nutrient absorption, reducing inflammation, and preserving IgA integrity for intestinal homeostasis [29,30,31,32]. In broilers, supplementation with the plant decreased *Clostridium perfringens*, *Escherichia coli*, and *Staphylococcus* while increasing *Lactobacillus*, *Bacteroides*, and *Firmicutes* [33,34,35,36]. As *Lactobacillus* produces short-chain fatty acids (SCFAs) that aid in pathogen resistance [37], this suggests a protective role in gut health. Additionally, *A. annua* essential oil reduces *Enterobacteriaceae* in intestinal contents [33]. In weaned piglets, *A. annua* increased *Bacteroidota* abundance and raised acetic and butyric acid levels, metabolites that prevent inflammation and inhibit pathogens [38,39,40]. These findings highlight *A. annua* as a natural promoter of gut microbial balance and intestinal health.

Beyond its effects on microbiota, *A. annua* has also been shown to strengthen the intestinal barrier. This is achieved through the upregulation of tight junction proteins, including Claudin-2 and Zonula Occludens-1, which are crucial for maintaining gut integrity. In broilers receiving *A. annua* extract, the expression of these proteins significantly increased, particularly in animals suffering from necrotic enteritis [41,42,43]. Similarly, in weaned piglets, *A. annua* residue, a byproduct of the industrial extraction of artemisinin, enhanced the expression of tight junction proteins [39].

In addition to supporting gut structure, *A. annua* exhibits potent anti-inflammatory properties. This is evidenced by a reduction in pro-inflammatory cytokines, such as IL-1β, IL-6, and TNF-α [36]. Supplementation with *A. annua* extract in chickens and weaned pigs significantly lowered IL-1β, IL-6, and TNF-α levels in jejum [39,43]. Furthermore, the plant enhanced both cellular and humoral immunity, providing better protection against infections. Its supplementation has been linked to increased levels of immune markers in chickens such as secretory IgA (sIgA), IgG, and IL-10, indicating a strengthened immune response [44,45]. Additionally, weaned pigs receiving *A. annua* residue showed higher levels of serum C3 and IgA, suggesting enhanced immune protection and reduced inflammation. C3, a key protein in the immune system, plays a crucial role in pathogen defense by marking microbes for destruction and regulating immune responses [39,46].

Research indicates that *A. annua* enhances growth performance in poultry. In broilers, administering *A. annua* water extract resulted in increased final body weight and improved feed efficiency [47]. By day 42, the supplementation improved the metabolic rates of dry matter, crude protein, and ether extract, while increasing digestive enzyme activity, including duodenal chymotrypsin, trypsin, and jejunal lipase [47]. Another notable effect was a reduction in fecal gas emissions, particularly ammonia (NH_3_) and hydrogen sulfide (H_2_S), which are major contributors to air pollution in modern poultry systems [47].

Interestingly, *A. annua* has also been shown to enhance resilience under stress and critical periods. Its supplementation supports post-weaning adaptation in pigs and improves stress tolerance in chickens. Pigs fed *A. annua* for 30 days showed reduced diarrhea, improved nutrient digestibility, and greater body weight gain [44]. In broilers under heat stress, *A. annua* powder and oil increased daily feed intake and weight gain [48]. Additionally, supplementation during pregnancy or lactation benefits offspring. Sows receiving *A. annua* extract had lower serum endotoxin, Heat Shock Protein 70 (HSP-70), and inflammatory cytokines, while showing higher serum triiodothyronine (T_3_) levels and feed intake [49,50].

Finally, recent research indicates that *A. annua* benefits also extend to rumen health. In lambs, a water extract of the plant enhanced immune function by increasing sIgA, IL-4, IL-2, and genes related to inflammation regulation and tissue repair while also improving antioxidant status by increasing Total Superoxide Dismutase (T-SOD) and Glutathione Peroxidase (GSH-Px), and reducing Malondialdehyde (MDA), a marker of oxidative stress [51]. In dairy cows, supplementation improved rumen fermentation by increasing bacteria linked to propionic acid production and reducing those associated with energy loss and acidosis [52,53]. It also boosted milk production, lactose percentage, immunity (↑IgM, ↓IL-1β), and antioxidant capacity (↑SOD, GSH-Px, ↓MDA) [53].

**Figure 1:**
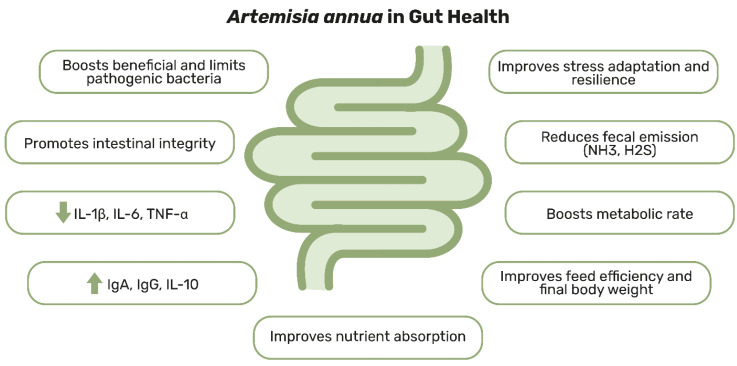
Schematic representation of the main effects of *Artemisia annua* on gut health in animals.

Within this evolving landscape, *A. annua* and its active compound, artemisinin, have demonstrated significant potential in supporting gastrointestinal and ruminal health, modulating the microbiome, and reducing inflammation. Their ability to regulate macrophage polarization, suppress pro-inflammatory cytokines, and restore epithelial integrity highlights their promise as therapeutic agents for IBD and other inflammatory gut disorders [54,55,56,57]. As previously mentioned, *A. annua* supplementation has been shown to enhance gut microbiota diversity, improve immune responses, and mitigate bacterial infections across multiple species. Given the complexity and individuality of the gut microbiome, further research is required to optimize the efficacy, safety, and appropriate dosages of *A. annua* and artemisinin in other animal species, such as companion animals. These findings underscore their potential in advancing gastrointestinal therapies, offering novel solutions for managing intestinal diseases while promoting microbiome balance and overall health. Figure 1 illustrates the key properties of *A. annua* and its therapeutic potential for gut health. All technical details related to the scientific publications cited in this section regarding the effects of *A. annua* and artemisinin on animals (including animal age, extract properties, and administered doses) are provided in Supplementary Table S1.

## 4. The Antiparasitic Potential of *Artemisia annua*

*A. annua*, widely recognized for its role in malaria treatment, has also demonstrated promising antiparasitic properties in animals. Its active compound, artemisinin, is a key component of artemisinin-based combination therapies (ACTs) against *Plasmodium* spp., the protozoan responsible for malaria. The same mechanism underlying its efficacy in human parasites (generating reactive oxygen species (ROS) that damage parasite cells) also applies to various animal parasites (*Leishmania* spp., *Trypanosoma* spp., *Eimeria* spp.) [58]. This similarity in action highlights *A. annua* as a potential natural alternative for managing parasitic infections in animals. The following section details key studies supporting its antiparasitic potential.

### 4.1. Coccidiosis

Coccidiosis is a major health issue affecting a wide range of animals, including livestock, poultry, and companion animals such as dogs, cats, and rabbits [59]. It poses a significant economic burden globally, leading to treatment costs, increased vulnerability to secondary infections, mortality, and decreased productivity [60]. For instance, annual global losses due to coccidiosis in poultry are estimated to exceed USD 14.5 billion [61].

Coccidiosis is caused by intracellular protozoa that primarily infect vertebrates, developing predominantly within the intestinal epithelial cells [62]. Symptoms of the infection can include diarrhea, weight loss, dehydration, and even death in extreme cases. The disease is highly contagious, spreading rapidly in environments with poor sanitation and overcrowding spaces. The life cycle of *Coccidia* includes both environmental and host-associated stages. It starts when infected hosts excrete oocysts, the parasite’s external form [62]. Under suitable conditions of air, moisture, and warmth, these oocysts undergo sporulation and become infective. Each oocyst contains four sporocysts, each with two sporozoites [63]. When ingested by a host, sporozoites emerge, invade intestinal cells, and develop into schizonts, which produce merozoites through asexual reproduction. These merozoites infect new cells, spreading the parasite. After several asexual cycles, sexual reproduction occurs, forming macrogametes and microgametes. Their fertilization creates a zygote, which matures into a new oocyst, continuing the cycle [62,63,64].

Coccidiosis, caused mainly by *Eimeria* species, significantly affects poultry and ruminants. In poultry, *E. acervulina* is the most prevalent in commercial flocks [62], followed by *E. tenella* and *E. maxima* [65]. The disease causes diarrhea, anemia, dehydration, lethargy, poor growth, reduced feed efficiency, and decreased production (e.g., egg or milk yield) [66,67,68,69]. Affected animals may also exhibit ruffled feathers (in poultry), weight loss, and general weakness [66]. In severe cases, the disease can lead to high mortality, particularly in young animals [67,68,69]. Severity depends on species, exposure, and immune status [68,70]. In ruminants, immunity typically develops within the first year, but adults can act as reservoirs [68].

Treatment and prevention of coccidiosis in both poultry and ruminants rely on anticoccidial drugs, primarily antibiotics [68,71]. However, misuse has led to drug resistance [72], prompting a search for alternative solutions. Plant-based options, such as herbal extracts, are gaining popularity as feed additives to enhance immunity and reduce drug residues in the food chain [73]. For instance, in Europe, the ban on antibiotic growth promoters has accelerated the transition toward natural feed additives; therefore, an increasing global demand for sustainable, safer animal products is further driving the adoption of plant-based solutions [74].

Among plant-based alternatives, *A. annua* and its primary active compound, artemisinin, have gained attention for their potential against coccidia in animals. Several studies have explored their effects, with the majority focusing on poultry, as chickens are most affected by coccidiosis. The potential of *A. annua* as a natural anticoccidial agent has been extensively studied. Diverse doses ranging from 0.5% to 5% *A. annua* in broiler reduced the severity of intestinal lesions, decreased *E. acervulina* and *E. tenella* oocyst shedding, and improved weight gain, villus height, and crypt depth, particularly in the jejunum and ileum [75,76,77,78,79]. Additionally, it modulated the expression of *IFN-γ* and *IL-10*, indicating a regulatory effect on immune responses [75]. Lastly, the feed conversion ratio (FCR), a key measure of feed efficiency and production performance, improved in highly and low-infected chickens with *E. tenella* and treated with the plant [76,80].

Beyond its therapeutic effects, *A. annua* has also shown significant prophylactic benefits in coccidiosis control. Chickens fed with *A. annua* experienced an 80% reduction in lesion scores and decreased oocyst counts, with *E. tennella* and *E. acervulina* being the predominant species found [81]. Similarly, in free-range broilers, *E. acervulina* oocyst output was reduced by 60–70% [82]. While complete parasite elimination is unlikely, *A. annua* helps reduce infection levels, enhances immune response, and improves flock resistance to coccidiosis. These findings position *A. annua* as a promising natural alternative for disease management [82].

The anticoccidial properties of *A. annua*, in both natural and fermented forms, have also been evaluated in other species, including lambs and rabbits. A study by Liu, S. et al. [83] in lambs found that all groups treated with the plant showed strong anticoccidial effects, with oocyst reduction and weight gain. Fermented *A. annua* in this species also reduced pro-inflammatory cytokines (IFN-γ, IL-1β, IL-17) while increasing the anti-inflammatory IL-10, suggesting broader protective effects against coccidiosis [84].

In rabbits infected with *Eimeria* spp., administration of *A. annua* extract significantly increased weight gain and reduced fecal oocyst shedding [85]. Moreover, rabbits receiving this dose exhibited the lowest total bacterial counts in their fecal content, while their total volatile fatty acids (VFAs) levels were the highest [85]. VFAs play a crucial role in digestive physiology and overall health in rabbits by supporting energy metabolism, gut microbiota balance, pH regulation, and overall growth performance [86]. Furthermore, incorporating *A. annua* powder into the diet resulted in improved growth performance and effective coccidiosis prevention [85].

The mode of action of *A. annua* against *coccidia* has begun to be elucidated, with artemisinin identified as a key active compound targeting oocysts and disrupting their formation by interfering with oocyst wall development. This leads to the death of oocyst and a reduced sporulation rate [87]. Oocyst sporulation plays a critical role in coccidiosis epidemiology, as animals are primarily infected by ingesting sporulated oocysts. During *Eimeria* infection, the NF-kB protein complex is activated to protect parasitized cells from apoptosis, allowing merozoites to mature. Subsequently, *Eimeria* inhibits NF-kB to facilitate host cell apoptosis and promote merozoites escape [88]. Artemisinin has been shown to counteract this process by promoting apoptosis in parasite-infected cecal cells by increasing caspase-3 activity, a key enzyme in apoptosis execution, while decreasing Bcl-2 levels, a protein that inhibits apoptosis [89]. Studies using whole-plant or dried-leaf extracts of *A. annua* have demonstrated the inhibition of oocysts sporulation, morphological changes in oocysts, and suppression of NF-kB expression [90,91].

Isolated artemisinin has also been explored against coccidia. Studies showed that artemisinin improved weight gain and the feed conversion ratio in broilers infected with *E. acervulina* and significantly reduced oocyst production in animals infected with mixed *Eimeria* species (*E. acervulina*, *E. tenella*, and *E. maxima*) [78,92]. While artemisinin appears to be the primary bioactive compound responsible for combating coccidiosis, other constituents of *A. annua*, such as camphor and 1,8-cineole, have also demonstrated anticoccidial activity [78].

Beyond its direct anticoccidial properties, *A. annua* also offers additional benefits that boost protection against coccidia. Broilers supplemented with *A. annua* exhibited improved feed conversion efficiency and greater weight gain compared to those fed with conventional diets [93]. These effects are likely due to the plant’s rich composition of crude protein, essential amino acids, minerals, vitamins, antioxidants, and flavonoids, all of which support growth and overall health [94]. Additionally, animals receiving *A. annua* supplementation showed improved reproductive performance, including increased egg production and larger egg sizes [75,93].

**Figure 2:**
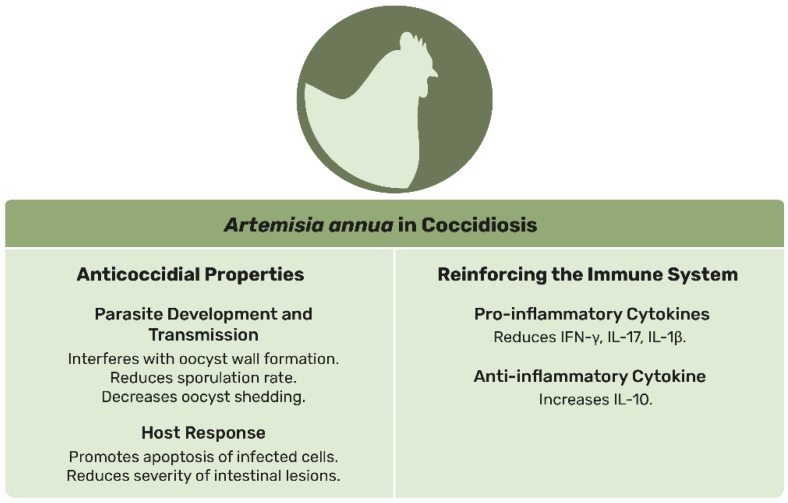
Schematic representation of the main effects of *Artemisia annua* on coccidiosis in animals.

In conclusion, *A. annua* and its active compound, artemisinin, show significant potential in managing coccidiosis, particularly in poultry. The plant has been shown to reduce oocyst output, improve feed conversion efficiency, enhance immune function, and promote growth and gut health. However, further research is needed to fully understand its long-term effects, optimal dosages, precise mechanisms of action across different species, and applicability in several species. Figure 2 highlights the key properties and therapeutic potential of *A. annua*, emphasizing its dual role as an anticoccidial agent and immune enhancer. As mentioned previously, all technical details related to the scientific publications cited in this section regarding the effects of *A. annua* and artemisinin on animals (including animal age, extract properties, and administered doses) are listed in Supplementary Table S1.

### 4.2. Leishmaniasis

Another important parasite for which the potential role of *A. annua* medicinal plant is beginning to be explored is *Leishmania* spp. Despite the fact that *Leishmania* can infect various animal species, companion animals, particularly dogs, are the most frequently affected. However, research regarding the efficacy of *A. annua* against this parasite in dogs and cats is still emerging, with most findings still coming from mouse models.

In dogs, the infection is primarily caused by the protozoa, *Leishmania infantum*. It is endemic in over 90 countries, with an estimated 700 million infected dogs, including 2.5 million cases in Europe [95,96]. Transmission mainly occurs via *Phlebotomus* (Old World) and *Lutzomyia* (New World) sandflies, though non-vectorial transmission (e.g., sexual, vertical, and blood transfusion routes) has also been reported [97,98]. Infected dogs exhibit variable clinical signs, often associated with immune complex deposition and excessive humoral responses [97,99]. While cats were traditionally considered less susceptible, they can contract *Leishmania infantum* and even transmit it to sandflies [100]. Feline leishmaniasis generally presents milder symptoms, but co-infections with immunosuppressive diseases can worsen clinical signs [97,100,101]. The increasing prevalence of Canine (CanL) and Feline (FeL) leishmaniasis highlights the urgent need for enhanced surveillance, climate-adaptive prevention strategies, and improved diagnostics and treatments to control this growing zoonotic threat.

The prevention and management of leishmaniasis in companion animals focus on minimizing sandfly exposure through insecticidal collars, sprays, and restricting outdoor activity during peak vector hours. Vaccination, though not fully effective, stimulates a protective Th1 immune response, reducing infection rates by 68.4% to 80%, but may interfere with serological diagnosis [102,103]. On the other hand, treatment focuses on controlling infection and alleviating symptoms through antiparasitic, immunomodulatory, and supportive therapies. Meglumine antimoniate and miltefosine aid parasite elimination, while allopurinol inhibits replication. Immunomodulators like dietary nucleotides and domperidone strengthen immune defenses, while supportive therapy helps manage complications [97,104]. However, complete cure remains elusive, with relapses within 5 to 12 months. Though standard treatments improve outcomes, they also pose risks such as nephrotoxicity, inflammation, and drug resistance, particularly to allopurinol [105,106]. The limitations of current treatments have driven research into alternative natural therapies, particularly plant-based solutions like *A. annua* and its main compound, artemisinin. Artemisinin generates cytotoxic ROS, exploiting *Leishmania*’s vulnerability to oxidative stress due to its lack of specific antioxidant enzymes [107,108,109].

Recent research indicates that artemisinin induces apoptotic-like cell death in *Leishmania*, primarily through mitochondrial alterations, leading to membrane potential loss, ATP depletion, and oxidative stress [110,111,112,113]. The efficacy of artemisinin is further enhanced by iron availability, which increases free radical generation and depletes non-protein thiols, critical components of the parasite’s antioxidative defense system [111,114].

Both in vitro and ex vivo studies demonstrate its broad-spectrum activity against multiple *Leishmania* species, highlighting its therapeutic potential and safety [110,111]. In vivo studies in infected mice further support artemisinin’s efficacy, with oral and topical treatments significantly reducing parasite burden, lesion size, and splenic weight in infections [111,112,115].

*A. annua* and its active compound, artemisinin, also exhibit significant immunomodulatory potential against *Leishmania*, promoting a shift toward a protective Th1 response. Artemisinin has been shown to restore macrophage nitric oxide production, which is essential for intracellular parasite elimination [111,116]. Additionally, it increases IFN-γ and IL-2 levels in infected mice to levels comparable to uninfected controls, reinforcing Th1 immunity [111]. Some studies also report elevated levels of both IFN-γ and IL-4, with a more pronounced increase in IFN-γ, further suggesting a Th1-biased immune response [112].

Whole-plant *A. annua* extracts may offer superior leishmanicidal and immunomodulatory benefits compared to isolated artemisinin, likely due to the synergistic effects of bioactive compounds such as diverse flavonoids, camphor, and β-caryophyllene [117,118,119,120]. Studies in *Leishmania donovani*-infected mice have shown that leaf extracts enhance immune responses by increasing IFN-γ while reducing IL-4 and IL-10 levels. Mice treated with *A. annua* also exhibited significantly higher nitric oxide production and increased CD4+ and CD8+ T-cell populations compared to pure artemisinin or amphotericin B treatments, highlighting the extra phytoingredient immunostimulatory potential [119]. Additionally, essential oils from *A. annua* have demonstrated complete clearance of *Leishmania* amastigotes in macrophages, with in vivo studies reporting significant parasite reduction in the spleen and liver [117,119].

Clinically, *A. annua* has shown promise in companion animals, particularly in cases where conventional treatments failed or caused adverse effects. A cat with cutaneous *Leishmania mexicana* lesions significantly improved after treatment with *A. annua* capsules, with stable lesions and no reported side effects after eight months [121]. Similarly, a dog with CanL treated with *A. annua* extract in combination with allopurinol exhibited marked clinical and laboratory improvements, including increased hematocrit levels and decreased Alpha-1, Alpha-2, and Gamma globulin levels, along with reduced IgG levels against *Leishmania* [122].

**Figure 3:**
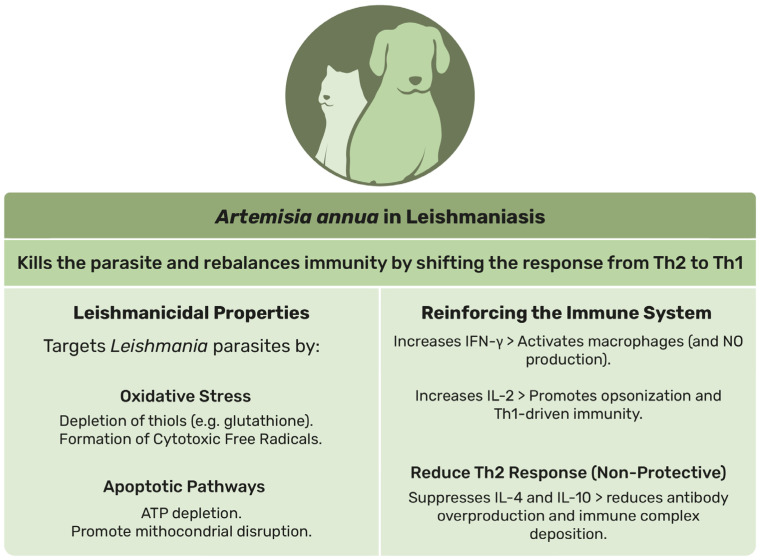
Schematic representation of the main effects of *Artemisia annua* on leishmaniasis in animals.

In summary, *A. annua* and artemisinin have demonstrated promising efficacy against *Leishmania* parasites, highlighting their potential as effective potential co-treatments alongside conventional therapies. However, further research is needed to optimize dosing protocols and assess long-term outcomes in companion animals. Figure 3 illustrates the key properties and therapeutic potential of *A. annua*, highlighting its dual role as a leishmanicidal agent and immune system enhancer. All technical data from the scientific publications cited in this section addressing the effect of *A. annua* and artemisinin not only in animals but also in vitro (given the limited number of in vivo studies) are also compiled in Supplementary Table S1.

### 4.3. Other Parasites

*A. annua* and artemisinin have also demonstrated promising antiparasitic effects against various pathogens affecting animal health, including *Toxoplasma gondii*, *Echinococcus multilocularis*, *Trichinella spiralis*, *Trypanosoma cruzi*, *Cytauxzoon felis*, and *Neospora caninum*. Studies indicate that *A. annua* can inhibit the intracellular replication of *Toxoplasma gondii* in vitro while also stimulating the immune response. Additionally, mice treated with the plant developed symptoms later than the control group [123]. Artemisinin and its derivatives have also been shown to interfere with parasite development by altering calcium dynamics [124,125,126].

Furthermore, *A. annua* and artemisinin inhibit the in vitro growth of *Trypanosoma cruzi* [127,128], *Cytauxzoon felis* [129], and *Neospora caninum* [130]. When combined with other compounds, artemisinin has also exhibited antiparasitic effects against *Echinococcus multilocularis* [131] and *Trichinella spiralis* [132].

All these findings suggest that both the plant and its active compound possess broad antiparasitic potential. Their effectiveness, whether used alone or in combination, underscores their promise for applications in veterinary parasitology.

## 5. *Artemisia annua*: A Green Hope in the War on Cancer

Cancer is one of the leading causes of death in mammals [133]. Like in humans, animals can develop a wide range of cancers, though their prevalence and characteristics vary depending on factors such as species, breed, age, and lifestyle [134]. Despite these differences, cancers share fundamental biological traits, known as the hallmarks of cancer, as described by Hanahan and Weinberg [135,136,137]. These hallmarks include sustained proliferative signaling, evasion of growth suppressors, resistance to cell death (apoptosis), induction of ferroptosis (a form of cell death caused by iron accumulation and oxidative stress), angiogenesis (formation of new blood vessels to supply tumors), activation of invasion and metastasis, deregulation of cellular energetics, immune evasion, conferring genome instability and mutation, and tumor-promoting inflammation [135,136,137].

Cancer research in animals is still in its early stages, and its occurrence across the entire animal kingdom remains underexplored. However, companion animals, particularly dogs and cats, are the most studied due to their close bond with humans, which has led to increased attention and economic interest, partly driven by the humanization of pets. Additionally, domesticated dogs and cats have experienced an increased incidence of cancer as medical and technological advances have extended their lifespans beyond what evolution had naturally prepared them for [138].

Currently, there is growing significant scientific interest in the anticancer potential of *A. annua* and artemisinin. Numerous in vitro, in vivo, and clinical studies suggest that artemisinin and other bioactive molecules in the plant, such as polyphenols, exhibit significant anticancer properties [139,140,141,142,143]. Furthermore, research indicates that these compounds may serve as adjuvants in cancer treatment, enhancing the efficacy of certain chemotherapeutic agents [144,145,146,147]. While most studies focus on human cancers or laboratory models such as mice, emerging evidence suggests that *A. annua* and artemisinin may also help reduce cancer cell proliferation in dogs and cats [148,149,150].

A study by Isani et al. (2019) [148] investigated the cytotoxic effects of pure artemisinin and a hydroalcoholic extract of *A. annua* on a canine osteosarcoma (OSA) cell line. The study found that both reduced cell viability (by increasing detached cells and cytoplasmic condensation), with the *A. annua* extract showing a stronger effect. Additionally, the IC50 values were eight times lower in *A. annua* extract in comparison with pure artemisinin, indicating a synergistic effect from additional compounds present in the plant.

Interestingly, the extract induced a sub-G1 population, indicating necrotic cell death rather than apoptosis, while also demonstrating reduced intracellular iron concentrations, suggesting that, similar to what occurs on humans and animals models like mice, artemisinin-induced cell death in other animal species may occur via ferroptosis, a programmed cell death mechanism associated with lipid peroxidation and oxidative stress [148,149,150,151]. Since cancer cells often exhibit high intracellular iron concentrations, they are more susceptible to ferroptosis. The endoperoxide structure of artemisinin enhances reactive oxygen species (ROS) production through interaction with Fe^2+^, intensifying ferroptotic mechanisms [151,152]. These findings suggest that the anticancer effects of *A. annua* in animals may be, at least in part, mediated by ferroptosis [153,154,155].

A subsequent study by Salaroli et al. (2022) [150] evaluated the effects of *A. annua* extract on additional canine osteosarcoma (OSA) cell lines (OSCA-8 and OSCA-40). The extract exhibited dose-dependent cytotoxicity, with significantly much lower IC50 values than pure artemisinin, reinforcing the idea that other bioactive compounds in the plant act synergistically. Additionally, an increase in intracellular iron content and lipid peroxidation was observed in cells treated with the extract, further supporting ferroptosis as the primary mechanism of cell death.

An in vivo study, though not focused on companion animal model cells, performed on mice with mammary tumors needs to be highlighted. The study demonstrated that artemisinin significantly reduces tumor proliferation by inhibiting angiogenesis. Its anticancer properties are linked to decreased vascular density and cell proliferation in breast and ovarian cancer xenografts [139]. Notably, artemisinin oil suspension led to a dose-dependent reduction in VEGF and HIF-1 serum levels, which play crucial roles in tumor progression, particularly in angiogenesis, with higher doses showing more pronounced effects [139]. Furthermore, *Notch1* expression, associated with breast cancer malignancy and metastasis, was downregulated in tumors treated with medium to high doses [139].

A study by Breuer at al. (2014) [156] examines the use of pulverized *A. annua* combined with iron as an adjuvant treatment for fibrosarcoma in companion animals (three dogs and one cat). Following surgical excision of the tumors, conventional therapy was complemented with *A. annua* supplementation. In all cases, *A. annua* exhibited a positive effect as an adjunct to surgery, with no observed tumor recurrence or new tumor development during the follow-up period [156]. These preliminary findings suggest the potential utility of *A. annua* as a supportive therapeutic agent in the management of fibrosarcoma in veterinary oncology. Following this study, a further one conducted by Saeed et al. (2019) [149] tested *A. annua* with iron as an adjuvant therapy alongside standard treatments in 16 dogs and 4 cats with various tumors. The study demonstrated that *A. annua* significantly improved survival rates, with 13 out of 20 treated animals surviving beyond 18 months, compared to none in the control group. The study assessed tumor characteristics and treatment response by measuring Transferrin Receptor (*TfR)*, a key protein for iron uptake, and *Ki-67*, a marker for cell proliferation. The results suggested that *A. annua* was more effective in aggressive tumors, where high expression of *TfR* and *Ki-67* correlated with better treatment response. Importantly, no significant adverse effects were observed in the treated animals [149].

**Figure 4:**
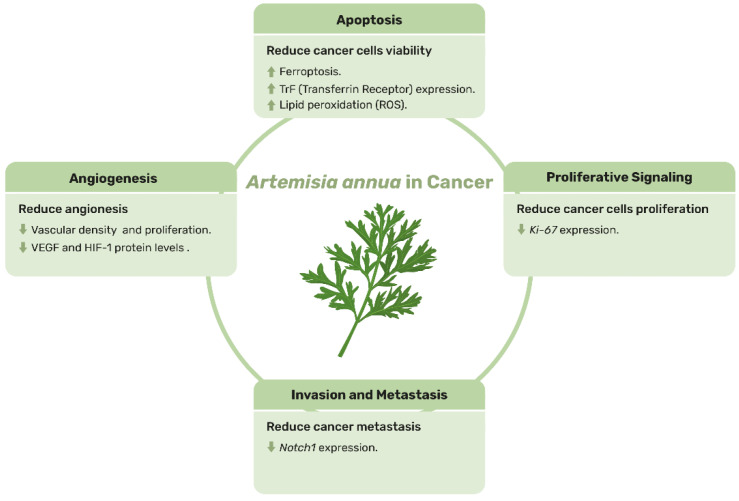
Schematic representation of the main effects of *Artemisia annua* on cancer in animals.

Despite these promising findings, further investigation is needed in both companion animals and livestock to establish the efficacy and safety of *A. annua* in cancer treatment. Rigorous clinical trials are essential to confirm these preliminary results, determine the optimal dosage and administration protocols, and explore its effects in a broader range of animal species and cancer types. A summary of the main hallmarks of cancer where *A. annua* and its bioactive compounds may play a beneficial role is presented in Figure 4, highlighting their potential as key components in future cancer treatment strategies. All detailed information from the scientific publications cited in this section, regarding the effects of *A. annua* and artemisinin not only in animals but also in vitro (due to the limited number of in vivo studies), is also presented in Supplementary Table S1.

## 6. Boosting the Effect: How Artemisinin Works in Synergy with Other Molecules

Pharmacodynamic synergy refers to how different chemical compounds present in the same plant interact to enhance or modulate the therapeutic effects of other compounds. This phenomenon is fundamental to professionalizing herbal medicine, as many medicinal plants contain a complex matrix of active substances that work together, as a polytherapy, and are often more effective than any single compound. Pharmacodynamic synergy in medicinal plants occurs through various mechanisms that include action on multiple targets such as improved bioavailability, potentiation of effect, or modulation of side effects. For instance, effect enhancers amplify pharmacological activity, as seen in *Hypericum perforatum*, where flavonoids and hypericins synergistically boost its antidepressant effects [157] or in green tea (*Camellia sinensis*), where catechins synergize with theine to improve antioxidant and thermogenic effects [158,159]. Additionally, synergies can also involve action on multiple targets, where compounds act on different pathways or receptors for a shared therapeutic goal as in *Curcuma longa* (turmeric), where curcumin’s anti-inflammatory and antioxidant properties are complemented by essential oils that enhance its bioavailability [160].

In the case of *A. annua*, the synergy between artemisinin and other biomolecules, such as flavonoids and other terpenes, has become a significant research focus. These compounds, found abundantly in the plant, may work together to enhance its therapeutic effects, potentially increasing its efficacy against various diseases [5]. Among the biomolecules present in *A. annua*, notable examples include terpenes, essential oils, phenolic compounds, and polysaccharides.

Terpenes are a significant class of biomolecules in *A. annua* with artemisinin as the main active compound. As mentioned above, artemisinin exhibits strong therapeutic activity by generating free radicals that damage parasites or induce ferroptosis in cancer cells. Beyond artemisinin, *A. annua* contains a wide variety of terpenes that contribute to the plant’s defense mechanisms and possess medicinal properties [7,161]. These terpenes include monoterpenes, sesquiterpenes, and diterpenes. Within the subcategory of monoterpenes, we find limonene, which gives the plant its characteristic aroma and exhibits antimicrobial and anti-inflammatory properties; pinene, known for its anti-inflammatory, antioxidant, and bronchodilator effects; and camphene, which possesses antioxidant and antifungal activity. Among the sesquiterpenes, we find not only artemisinin, but also artemisia ketone, which has antimicrobial and antioxidant activity; and β-caryophyllene, recognized for its anti-inflammatory and anxiolytic properties [7,161]. In general, the high variety of terpenes in *A. annua* enhances their synergy with other metabolites, contributing to a broad range of biological effects [7].

Another important group of compounds found in *A. annua* are the essential oils, a complex mixture of volatile compounds that contribute to the plant’s therapeutic properties. These essential oils are rich in terpenes, such as 1,8-Cineole (eucalyptol), with antimicrobial and expectorant properties; thymol, which exhibits antifungal and antioxidant activity; α-pinene and β-pinene, known for their antioxidant and anti-inflammatory properties; limonene, with its ability to fight pathogens and antioxidant effects; and β-caryophyllene, for its anti-inflammatory potential [162]. Unique to *A. annua*, artemisia ketone and chamazulene contribute antioxidant, antimicrobial, or potent anti-inflammatory activities. Additionally, non-terpenic compounds such as terpinen-4-ol, which has antimicrobial properties and promotes healing, and bornyl acetate, known for its soothing and anti-inflammatory effects, also enhance the biological activities of these oils [163,164].

*A. annua* also contains phenolic compounds, which are produced by plants as a defense mechanism against diseases and stress. These compounds significantly contribute to the plant’s therapeutic properties, particularly its antioxidant and anti-inflammatory properties. Phenolic compounds are secondary metabolites, including flavonoids, phenolic acids, and coumarin derivatives, known for their high capacity to neutralize free radicals and regulate inflammatory processes [6]. Among these secondary metabolites, flavonoids are particularly notable. The leaves of *A. annua* contain several important flavonoids, including casticin, which has anti-inflammatory, antioxidant, antitumor, neuroprotective, and analgesic effects; quercetin, a potent antioxidant and anti-inflammatory agent; eupatorin, which exhibits antitumor, antioxidant, anti-inflammatory, antimicrobial, and cardioprotective effects; and artemetin, a flavonol with neuroprotective, antioxidant, and antitumor properties [6,165,166,167].

Flavonoids not only contribute to the plant’s biological activities but may also enhance the bioactivity of artemisinin. For example, flavonoids can facilitate the conversion of iron forms, aiding in the release of free radicals and improving artemisinin’s bioavailability and longevity in the body [168,169]. While casticin and artemetin alone show no antiparasitic activity, they synergize with artemisinin to boost its efficacy. Quercetin, on the other hand, possesses intrinsic antiparasitic activity that is further enhanced when combined with artemisinin [5]. In addition to flavonoids, *A. annua* contains coumarins, such as scopoletin and esculetin, that offer hepatoprotective and immune-modulating effects [6,167].

To conclude, it is important to highlight the role of polysaccharides, which have gained increasing attention in recent years due to their immunomodulatory, antioxidant, and antitumor properties [51,170]. These compounds interact with the immune system, contributing significantly to the therapeutic activity of *A. annua*. The polysaccharides in this medicinal plant include heteropolysaccharides composed of various monosaccharides, such as glucose, mannose, galactose, xylose, and arabinose. These compounds often feature β-glucan bonds and side branches that confer specific immunomodulatory and antioxidant properties. Other types of polysaccharides include sulfated polysaccharides, which demonstrate antioxidant and anticancer activities, and arabinogalactans, branched glycans with immunostimulatory effects that activate macrophages and enhance cytokine secretion. Furthermore, glucans, such as β-glucans, are well known for their ability to stimulate immune responses [171]. Some *A. annua* polysaccharides have been found to modulate immune responses by promoting the production of nitric oxide (NO), a critical mediator in immune defense. Additionally, these polysaccharides function as prebiotics, improving the composition of the intestinal microbiota, which may enhance the body’s resistance to infections [36,51,170]. These multifaceted properties underscore the importance of polysaccharides in *A. annua’s* therapeutic potential.

Taking into account the presence of these molecules in *A. annua* leaves and their therapeutic potential, it is crucial to highlight the pharmacodynamic synergy within this plant. This synergy enhances artemisinin’s efficacy by addressing its relatively short half-life in the body and improving its bioavailability (i.e., the ability of a molecule to be absorbed into the bloodstream) [172,173]. Although further research is needed, interesting studies have been released describing potential synergies, especially for flavonoids, which play a pivotal role, not only by increasing artemisinin’s bioavailability but also by enhancing its efficacy. They achieve this by targeting the same parasites through distinct biochemical pathways, thereby amplifying the therapeutic effect through synergistic mechanisms [5,173].

This synergy is mainly facilitated by bioactive compounds such as flavonoids and polysaccharides, which improve intestinal membrane permeability and consequently absorption, [36,174]. In addition, they influence metabolism by inhibiting liver enzymes, such as cytochrome P450, reducing artemisinin degradation and prolonging its activity in the body [175,176,177], as well as by increasing artemisinin’s availability at its site of action by interacting with cellular transporters, therefore amplifying its therapeutic effects [176,178]. Flavonoids like casticin and quercetin can also protect artemisinin from oxidative degradation, a key mechanism responsible for drug instability, thereby enhancing its stability and effectiveness during treatment [172,179]. Notably, studies have shown that combining artemisinin with flavonoids can reduce parasite load more effectively than artemisinin alone [162,178].

Research confirms the superiority of *A. annua* as a polytherapy over artemisinin monotherapy. A study by Weathers (2023) [169] demonstrated that orally administered dried *A. annua* leaves resulted in 45 times higher serum artemisinin levels and significantly greater artemisinin concentrations in the bloodstream compared to pure artemisinin at the same dose in mice, indicating enhanced bioavailability. Similarly, Desrosiers et al. (2020) [168] examined artemisinin bioavailability by comparing hepatic metabolism, tissue distribution, and inflammation attenuation between *A. annua* and semi-synthetic artemisinin in mice. Their findings showed that artemisinin from *A. annua* leaves exhibited superior anti-inflammatory potency, greater bioavailability, and broader tissue distribution (heart, lungs, liver, spleen, muscle, brain) than semi-synthetic artemisinin at the same artemisinin concentration, just one hour after administration. This increased efficacy is attributed to the presence of other *A. annua* phytochemicals that synergistically enhance artemisinin bioavailability by inhibiting liver P450s enzymes (CYP2B6, CYP3A4), preventing artemisinin degradation, and exerting anti-inflammatory effects that further optimize its therapeutic potential [168].

**Figure 5:**
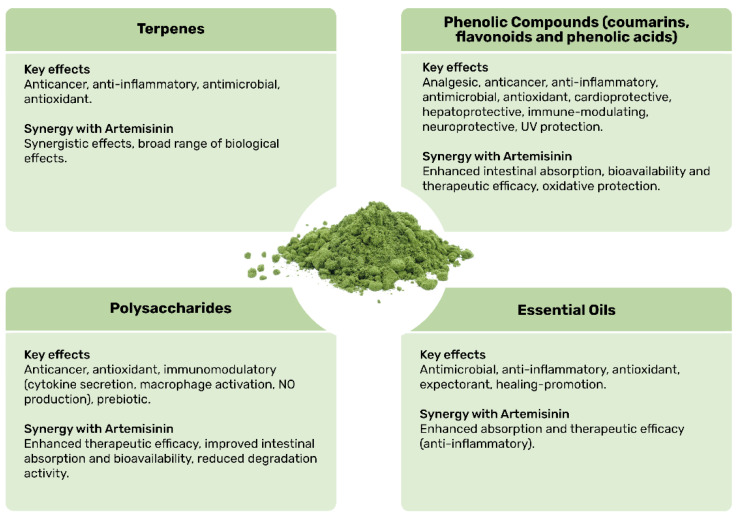
Schematic summary of the main compounds, and their key effects and synergy with artemisinin, identified in *Artemisia annua*.

Beyond malaria, the compounds in *A. annua* enhance artemisinin’s activity against diverse conditions, including parasitic infections, cancer, bacterial, and viral diseases [165,168,177]. This evidence underscores the promise of integrative medicinal approaches that leverage the pharmacodynamic synergy of *A. annua*. Continued research aims to refine formulations that maximize this synergy, offering innovative strategies for treating infectious and chronic diseases. A summary of the main compounds identified in *A. annua* and their synergistic effects with artemisinin is provided in Figure 5.

## 7. The Key to Quality: The Role of Standardization in Medicinal Plants Like *Artemisia annua*

All medicinal plants contain a variety of bioactive compounds or active ingredients that are responsible for their therapeutic effects. However, if these active ingredients are not standardized, variations in the concentration of these compounds can lead to unpredictable results, potentially reducing treatment efficacy or causing unwanted side effects. It is therefore essential to standardize medicinal plants in terms of active ingredient content in order to ensure the efficacy, safety, and quality of a product based on medicinal plants [180,181,182,183,184,185]. In fact, standardization is a key step in complying with international regulations and standards for the quality and safety of natural products. This facilitates the inclusion of medicinal plants in pharmacopeias and conventional medical practice [186].

This principle should be directly applied to *A. annua*, where artemisinin is the primary active compound [187,188]. However, the artemisinin content in the plant can vary significantly due to multiple factors, including the plant variety or chemotype (with low-, medium-, and high-artemisinin content varieties); growing conditions (such as soil, climate, and photoperiod); harvest timing (as artemisinin concentrations peak during the flower induction phase); and post-harvest processing of the plant (including drying methods and storage conditions) [189,190,191,192,193]. These factors directly influence the quality and consistency of the final product, affecting both the artemisinin levels and the presence of other bioactive compounds, such as flavonoids, which contribute to pharmacodynamic synergy. Given these variations, standardizing the artemisinin content within defined thresholds is crucial to ensure therapeutic potency and clinical efficacy, preventing both under- and overdosing. Without such standardization, the effectiveness of *A. annua* in treating diseases and other potential applications could become inconsistent, limiting its clinical reliability.

Studies on various *A. annua*-based products have revealed significant variability in artemisinin content, with some concerning differences. A recently published small-case study [194] analyzed four different artemisinin supplements marketed for dogs in the USA, finding that none of the products met acceptable strength ranges. The results showed high variability in artemisinin content compared to their labeled claims. Alarmingly, one of the products contained no detectable artemisinin, raising critical concerns about its efficacy. Additionally, stability testing and impurity analysis were not conducted, meaning that none of the products met the United States Pharmacopeia (USP) or ICH acceptance criteria [194]. Such inconsistencies found in animal health products highlight the need for rigorous quality control in plant-derived medicinal treatments.

Standardization is not only crucial for ensuring consistency and quality, but also for accurately assessing the toxicity levels of the active compounds. Although no scientific evidence has been generated using *A. annua* specifically as a phytoingredient, some studies in rats indicate that oral administration of *A. annua* extracts generally results in relatively high LD_50_ values, suggesting low overall toxicity [195]. For instance, an oral dose of 5000 mg/kg in mice produced no adverse effects, supporting the safety of oral administration as a route of delivery [196].

Additionally, the toxicity levels of *A. annua* toxicity should be directly applied to artemisinin, its primary active compound. No serious adverse effects have been reported from the oral administration of artemisinin. LD_50_ values in mice have been documented between 4228 and 5105 mg/kg when administered orally [197]. In humans, oral doses of 500 mg per day for five days have shown no adverse effects [198]. Considering that the artemisinin content in *A. annua* plant ranges from 0.01% to 1.0%, using the plant as a phytoingredient would result in the ingestion of a safe and well-tolerated amount of artemisinin [199,200].

Ensuring the standardization of *A. annua*’s active compounds, particularly artemisinin, is crucial to guarantee the efficacy, safety, and overall product quality. Proper standardization ensures batch-to-batch consistency, guaranteeing that each batch meets established quality standards. This is essential to provide reliable and effective treatments, protecting both consumer trust and patient safety. Without adequate standardization, the therapeutic potential of *A. annua* could be compromised, potentially endangering the health of those who rely on it.

## 8. Future Perspectives

This review highlights the promising therapeutic potential of *A. annua* and its active compound, artemisinin, in treating a wide range of health conditions affecting various animal species. Despite encouraging findings, further research is imperative to refine its therapeutic applications and fully establish its safety and efficacy. Continued investigation is necessary to optimize and standardize its use in future treatment protocols, ensuring the development of proper dosing guidelines and addressing concerns regarding potential adverse effects [201].

Moreover, there is a pressing need for additional studies to explore the compound’s effectiveness across different diseases, including its potential antiviral and antifungal properties [202,203,204], as well as its efficacy in diverse animal species. Research should also focus on investigating possible synergistic effects between artemisinin and other bioactive compounds within *A. annua* [146,205], improving its bioavailability [206,207,208], and further elucidating its mechanisms of action [208]. Such efforts are crucial for advancing the professional use of medicinal plants like *A. annua* as feed additives in animal health, thereby promoting the therapeutic applications of these medicinal plants and their bioactive components in veterinary medicine.

Additionally, the future success of *A. annua* as a feed additive will depend not only on its quality but also on its availability and affordability. Several factors, including production scale, cultivation methods, and regional agricultural conditions, influence both aspects. *A. annua* is relatively widespread and can be cultivated in many regions around the world with suitable climates, particularly in tropical and subtropical areas. Although the plant is not particularly difficult to grow, it requires specific optimal conditions, which can pose a significant challenge for achieving both high leaf biomass and adequate concentrations of therapeutic biomolecules.

In recent years, the cultivation of *A. annua* has gained increasing attention beyond its traditional use for artemisinin extraction to treat malaria in humans, moving into new fields such as human and veterinary medicine, as well as cosmetics. Nevertheless, a critical limitation remains: the current cost of production for animal health applications, especially for livestock. Large-scale commercial production of *A. annua* is still under development and has yet to achieve cost-effectiveness in most countries. However, as demand from the veterinary industry grows and cultivation and extraction technologies improve, it is expected that prices will decrease significantly, making *A. annua* a more accessible and viable option for animal health applications in the near future.

## 9. Conclusions

*A. annua* has a long history in traditional medicine, but its global significance grew in the mid-20th century with the discovery of artemisinin, a major breakthrough in malaria treatment that has saved millions of lives. This achievement earned Tu YouYou the Nobel Prize in Medicine in 2015 and highlighted the value of traditional knowledge and natural compounds in modern scientific research. Today, *A. annua* and its primary active compound, artemisinin, are at the forefront of research for their broad therapeutic potential in both human and veterinary medicine. In animals, current studies emphasize its effectiveness as a feed additive, promoting gut health, enhancing immunity, and aiding in the treatment of parasitic and cancer-related conditions.

*A. annua* and its bioactive constituents support intestinal health and animal growth by encouraging beneficial gut bacteria and suppressing harmful pathogens, improving microbial balance and reducing infection risk. It also demonstrates strong preventive effects against infections such as *Eimeria* spp. and *Clostridium perfringens*, particularly in poultry. Its rich content of flavonoids and antioxidants helps preserve intestinal integrity and boost immune function. Supplementation has been linked to improved egg production, feed efficiency, and survival in coccidiosis-infected birds. Higher doses have shown enhanced weight gain and reduced disease severity without adverse effects.

In companion animals, *A. annua* appears to be a safe and effective adjunct therapy for leishmaniasis, improving overall health and reducing parasite loads through artemisinin’s action, which promotes apoptosis and modulates immune responses. It also shows promise as an adjuvant in veterinary oncology, where whole-plant extracts, especially when combined with iron and conventional treatments, have extended survival and prevented tumor recurrence. This anticancer activity is likely linked to ROS-induced ferroptosis and enhanced by synergistic phytochemicals.

Overall, *A. annua* and its bioactive compounds, especially artemisinin, hold strong potential as feed additives to support animal health in treating gut issues, parasitic infections, and cancers in animals. However, inconsistent extract characterization across studies limits comparability.

Research into *A. annua* has expanded beyond artemisinin to include other bioactive compounds like flavonoids, phenolic acids, and polysaccharides, which work synergistically to boost artemisinin’s effectiveness and add their own therapeutic benefits. These interactions support the use of whole-plant extracts, positioning *A. annua* as a multi-target, polypharmacological agent ideal for complex disease treatment, especially in veterinary medicine. However, to realize its full potential, challenges such as variability in plant composition and lack of standardization must be addressed. *A. annua* exemplifies the integration of traditional knowledge with modern science and aligns with the “One Health” approach that connects human, animal, and environmental well-being.

Ongoing research is vital to unlock the full therapeutic potential of *A. annua* in veterinary medicine, including refining dosages, understanding bioavailability and safety, and tailoring treatments for different animal species. These efforts aim to enhance animal health, support personalized care, and promote more sustainable, evidence-based veterinary practices.

## Data Availability

Not applicable.

## Supplementary Materials

The following supporting information can be downloaded at: https://www.mdpi.com/article/10.3390/ani15101359/s1, Table S1: Detailed Data from In Vitro and In Vivo Studies on the Effects of *A. annua* and Artemisinin.
